# MX2: a high-flux undulator microfocus beamline serving both the chemical and macromolecular crystallography communities at the Australian Synchrotron

**DOI:** 10.1107/S1600577518003120

**Published:** 2018-04-03

**Authors:** David Aragão, Jun Aishima, Hima Cherukuvada, Robert Clarken, Mark Clift, Nathan Philip Cowieson, Daniel Jesper Ericsson, Christine L. Gee, Sofia Macedo, Nathan Mudie, Santosh Panjikar, Jason Roy Price, Alan Riboldi-Tunnicliffe, Robert Rostan, Rachel Williamson, Thomas Tudor Caradoc-Davies

**Affiliations:** a Australian Synchrotron, ANSTO, 800 Blackburn Road, Clayton, VIC 3168, Australia; b ARC Centre of Excellence in Advanced Molecular Imaging, Monash University, Clayton Campus, Clayton, Victoria 3168, Australia; c B21 SAXS, Diamond Light Source Ltd, Hartwell Science and Innovation Campus, Didcot OX11 0DE, England; d Department of Molecular and Cell Biology, University of California, Berkeley, Berkeley, California, USA; e California Institute for Quantitative Biosciences, University of California, Berkeley, Berkeley, California, USA; f Howard Hughes Medical Institute, University of California, Berkeley, Berkeley, California, USA

**Keywords:** microfocus beamlines, apertures, undulators, macromolecular crystallography, remote access, anomalous scattering, EIGER detector, long wavelengths, collimators

## Abstract

MX2 is an in-vacuum undulator-based crystallography beamline at the 3 GeV Australian Synchrotron. The beamline delivers hard X-rays in the energy range 4.8–21 keV to a focal spot of 22 × 12 µm FWHM (H × V). At 13 keV the flux at the sample is 3.4 × 10^12^ photons s^−1^. The beamline endstation allows robotic handling of cryogenic samples *via* an updated SSRL SAM robot. This beamline is ideal for weakly diffracting hard-to-crystallize proteins, virus particles, protein assemblies and nucleic acids as well as smaller molecules such as inorganic catalysts and organic drug molecules. The beamline is now mature and has enjoyed a full user program for the last nine years. This paper describes the beamline status, plans for its future and some recent scientific highlights.

## Abstract 2

A microfocus macromolecular crystallography beamline at the Australian Synchrotron is presented.

## 1. Introduction   

The macromolecular crystallography beamline (MX2) at the Australian Synchrotron complements the sister bending-magnet-based beamline (MX1) (Cowieson *et al.*, 2015 ▸) by catering to a compromise of the following two, not necessarily overlapping, needs of the community: high-flux crystallography for poorly diffracting crystals and micro-crystal crystallography for samples where matching beam size to sample size is critical to reduce background scattering. To achieve these aims the beamline has an in-vacuum undulator source in the 3 GeV storage ring of the Australian Synchrotron (Boldeman & Einfeld, 2004 ▸) producing a flux at the sample of 3.4 × 10^12^ photons s^−1^ (Owen *et al.*, 2009 ▸). After the double-crystal monochromator (DCM), a vertical focusing mirror (VFM) and two horizontal focusing mirrors (HFMs) produce a beam of 22 × 12 µm (FWHM H × V) at the sample position. The energy range of the beamline is 4.8–21 keV with a user changeable range from 8.0 to 15.5 keV (see Table 1 ▸). The experimental hutch is equipped with a sample changing robot (Cohen *et al.*, 2002 ▸, 2005 ▸; Russi *et al.*, 2016 ▸), a cryostream, an 8× zoom camera and a 311 × 327.8 mm EIGER X 16M pixel detector (Dectris Ltd, Baden-Dättwil, Switzerland), which recently (February 2017) replaced a Quantum 315r [Advanced Data Systems Corporation (ADSC), Paramus, NJ, USA].

MX2 first commenced user experiments in late 2008 and continues to run a fully subscribed user program with most beam time being used by the structural biology community. This community has been depositing to the Protein Data Bank (PDB) at an increasingly fast pace, reaching 191 PDB depositions in 2015 alone, with a total of 1766 so far [http://biosync.sbkb.org/ (accessed on 28 March 2018)]. During this time, the chemical crystallography community has made increasing use of the beamline, with this community presently accounting for around 10% of the total MX2 available time.

## 2. Beamline overview   

Where possible, the MX2 beamline uses identical equipment and software controls as its sister station MX1. Both beamlines underwent a significant software controls and computing hardware restructuring in recent years consisting of (i) introduction of a modular Python midlayer between the low-level Experimental Physics and Industrial Control System (EPICS) (http://www.aps.anl.gov/epics/) hardware control and the user interactive qeGUI (graphical user interface) (https://qtepics.github.io/) or *Blu-Ice* based GUIs; (ii) migration whenever possible of most Linux servers to virtual machines and Linux containers (LXC) running under Proxmox VE (https://www.proxmox.com). This has made MX2 easier to support and develop further. MX2, in particular, had several important performance and stability hardware upgrades in the last five years; namely, a replacement of the VFM substrate, introduction of a channel-cut Si(111) crystal [replacing a Si(311) double crystal] within the monochromator, replacement of the DCM water lines by electrical heating, replacement of the DCM cryocooler, replacement of the sample mounting robot, introduction of two attenuator wheels, replacement of the goniometer, a new sample view zoom camera, and most recently replacement of the detector. Finally, data storage was made more resilient by having two copies (one onsite and one offsite) and by compressing each experiment into a squashFS read-only file system.

### 2.1. Optics hardware and design   

**Figure 1:**
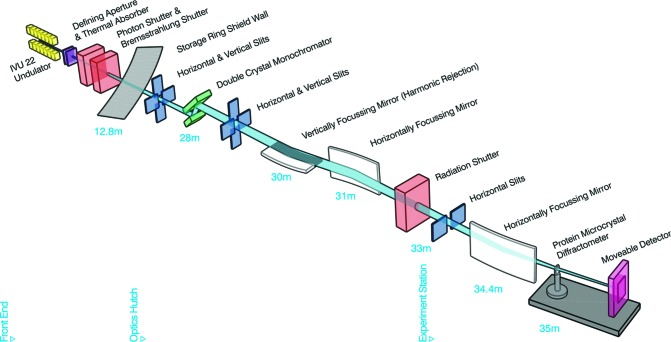
Schematic layout of the MX2 beamline. Components are undulator source (yellow), beam defining masks (purple), safety shutters (peach), slits (blue), mirrors (white), monochromator (green), goniometer (grey) and pixel array detector (pink). Distances are metres from the source.

A schematic diagram of the beamline is shown in Fig. 1 ▸ with the configuration of its optical elements, main beam characteristics and endstation equipment summarized in Table 1 ▸. The Australian Synchrotron SR03 straight section is equipped with a 3 m U22 in-vacuum undulator that serves as X-ray source (Fig. 1 ▸) for the MX2 beamline. Two separate liquid-nitro­gen-cooled Si(111) crystals on the DCM vessel situated in the optics hutch, 28 m from the primary source and 7 m from the sample (Fig. 1 ▸), can be used for energy selection. Within the DCM vessel, a double Si(111) crystal system produces a beam with a broad energy range [4.8 to 21.0 keV (2.58 to 0.59 Å)], whilst the channel-cut Si(111) produces a beam with a narrower energy range of 12.0 to 15 keV with reduced beam position and intensity variation and higher resilience to external vibrations. The latter is particularly suitable for aperture-based micro-beam work where beam stability is crucial. Downstream of the DCM there is a 300 mm eight-element bimorph VFM and a mechanically bent 700 mm HFM. Harmonic rejection is achieved by using Si or the Rh and Pt stripes on all mirrors. Three fluorescence screens with CCD cameras are strategically located along the beam path to allow beam alignment after shutdown periods. In the endstation, at 34.4 m from the source, there is a second horizontal microfocusing mirror (MHFM). This 400 mm 12-element bimorph mirror works as an additional horizontal focusing element reducing the beam size from ∼1.1 mm to below 20 µm (H) at the sample position.

### 2.2. Endstation hardware and design   

The endstation equipment includes a helium purged assembly that houses the fast shutter, two attenuator wheels and an aperture-based beam size selector device. The remaining hardware in the endstation consists of a sample camera, beamstop, photodiode, goniometer, cryostream system, detector, sample changer and a fluorescence detector.

A fast rotary beam shutter is located upstream of the sample. Beam steering and positional feedback is provided by optical visualization of the beam on a neodymium-doped yttrium aluminium garnet (YAG) crystal attached to the fast shutter (MacDowell *et al.*, 2004 ▸). Briefly, a 6 mm-diameter stainless steel cylinder rotates to allow the beam to pass through a small slot cut through the cylinder. When the shutter closes, a YAG crystal rotates into the path of the beam generating visible light. This light is then reflected *via* a small prism to a CCD camera (Point Grey Research Inc., Richmond, BC, Canada) which records the live image of the beam. The centroid of the beam image is calculated from this input using *areaDetector* (Rivers, 2010 ▸). The beam is steered to a reference position by a proportional-integral-derivative slow-feedback loop using piezo motors on the VFM pitch for vertical steering and on the HFM for horizontal steering. The steering system becomes active each time the shutter is closed.

The attenuator assembly was designed to achieve fine-tuning of attenuation across the available energy range. This includes the long wavelengths needed for sulfur single-wavelength anomalous diffraction (SAD) as well as the short wavelengths needed for high-resolution chemical crystallography (better than 0.7 Å at the detector edge). This assembly consists of two discs (attenuator wheels), each one cut with a taper for half of the disc with a set of apertures on the other half. The apertures are either left open or covered with various thicknesses of aluminium foil or graphite. The thickness of the tapers varies from 3.00 to 0.010 mm and 1.50 to 0.050 mm for the aluminium and the graphite wheels, respectively. The use of the graphite taper in the long-wavelength range and the aluminium taper in the short-wavelength range ensures an almost continuous range of attenuation from 0.01 to 100% transmission.

The beam aperture assembly is located 65 mm from the sample, and consists of a plate with a quincunx geometric hole pattern that is motorized vertically and horizontally by a pair of piezo motors (attocube; Munich, Germany). Four of the five holes are fitted with an electron microscopy aperture (centre aligned with the beam path). The apertures are made in 95:5 platinum:iridium (Ted Pella Inc, Redding, CA, USA) with dimensions 2.0  × 0.6 mm (outer diameter × thickness). In the default setup, a 1.0 mm centre hole provides full beam, and apertures with sizes of 300, 20, 10 and 7.5 µm are installed in the remaining positions. A forward scatter guard consisting of a 0.6 × 20 mm (inner diameter × length) tungsten tube is located downstream of the apertures.

**Figure 2:**
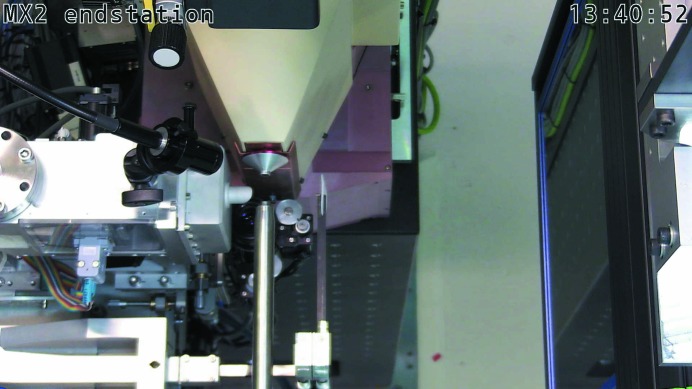
MX2 sample environment showing the rotation axis (middle top); the cryostream and illuminated back-stop projecting in from middle bottom; the sample light projecting in from left to the sample and the EIGER X 16M detector on the right.

The beam exits the beamline through a small aperture cut through a mirror mounted on the final nose piece. The mirror held at 45° to the beam direction allows visualization of the sample (from the beam direction) by a camera (Point Grey Research Inc., Richmond, BC, Canada) mounted perpendicular to the beam (Fig. 2 ▸). This setup allows a view of the sample along the beam axis with the camera allowing a variable magnification of between 2× and 8× (Navitar, Rochester, NY, USA). A cold LED-based sample light (SugarCube) is focused on a disc of thin white packing foam mounted on the beam stop. The foam is transparent to X-rays and allows the sample to be back-lit without the need for a light source that must move out of the direct beam each time a diffraction image is measured (MacDowell *et al.*, 2004 ▸). The use of a foam back-light allows an optical view of the sample to be continuously available during data collection (a still image of the sample is recorded before each collection). The 3 × 5 mm (diameter × length) beamstop is mounted on a thin carbon fibre arm that is held in place *via* a pneumatically actuated flipper. A second flipper holds a photodiode (which allows for flux intensity measurements). The photodiode is connected to a Keithley 6487 picoammeter (Keithley Instruments, Ohio, USA), allowing high sampling and recording of data. While not usually in the beam path, the photodiode can easily be moved into position for data acquisition, allowing regular maximum flux, energy range attenuator wheel calibrations or knife-edge beam size measurements.

The goniometer that was designed and built in collaboration with Stanford Synchrotron Radiation Lightsource (SSRL) contains an ABRS-250MP air bearing (Aerotech Inc, Pittsburgh, PA, USA) and rotates the sample around a horizontal axis. Two piezo-driven linear motors (SLC-2430, SmarAct GmbH, Oldenburg, Germany) centre the sample on the rotation axis with a sphere of confusion better than 1 µm (Figure S1 in the supporting information). The sample centring motors and goniometer post are protected from the cryostream by a small aluminium conical heater shield and a larger copper shield with heating elements. This assembly is further protected from endstation temperature variations by a three-dimensional printed Renshape cover.

The sample temperature is controlled by a cryostream (Cryojet 5, Oxford Instruments, Abingdon, UK) mounted on-axis with the sample pin. This system maintains the required temperature at the sample position. Temperature at the sample can be varied between 85 and 500 K. An interface for remote temperature control has been developed in-house using EPICS and the instrument serial communication. This allows easy temperature and flux control including automation of collections of the same sample at different temperatures for material science investigations.

An EIGER X 16M is mounted on an A-frame allowing a sample-to-detector distance of between 108 and 800 mm. The detector is protected from mechanical damage by an automated rolling cover (ADS GmbH) which is interlocked to the search of the hutch. In addition, a laser curtain system (S300, SICK) is installed preventing the forward movement of the detector if an obstacle is detected. The software setup includes a low-level EPICS layer running *areaDetector* (Rivers, 2010 ▸) and the AD-EIGER driver (https://github.com/brunoseivam/ADEiger) but also Python as a midlayer between the user GUI and the EPICS layer for the logic sequencing. Triggering is done *via* hardware from the goniometer motion controller using the air-bearing as the source.

Samples can be mounted manually or *via* an SSRL automated mounting system (SAM) (Cohen *et al.*, 2002 ▸, 2005 ▸; Russi *et al.*, 2016 ▸). The SAM supports the use of SSRL-style cassettes or Unipucks allowing for remote data collection by our user community. Typically, the robot (Cohen *et al.*, 2002 ▸) can exchange samples in approximately 3 min. MX2 uses the *Blu-Ice* control system (McPhillips *et al.*, 2002 ▸) with a newly mounted crystal ready for data collection using ‘click-to-centre’ sample centring in 15–30 s.

A sensitive silicon-drift fluorescence detector (Vortex 90EX, Hitachi High-Technologies Science America, Chatsworth, CA, USA) is automatically moved to an optimal position a few millimetres from the sample (*via* an in-house design consisting of a rodless pneumatic actuator and housing) for anomalous scattering energy selection experiments (called MAD scans in *Blu-Ice*) as well as for heavy-element identification experiments (called excitation scans in *Blu-Ice*).

### 2.3. Experimental parameters and analysis   

The size of the beam at the sample position is approximately 22 × 12 µm in diameter (FWHM, H × V). In general, this beam size allows for routine collection of data from crystals with dimensions larger than 10 µm for protein samples but has achieved excellent results for chemical and viral crystals with dimensions smaller than 5 µm. Typically diffraction data are collected in 18 s with a non-attenuated beam for a 180° data set with the detector set at 100 Hz frame rate.

Visiting researchers can easily change energy from 8.0 to 15.5 keV, and with staff intervention a larger range from 4.8 to 21.0 keV is available but will require a change of mirror stripe as well as tweaking to realign the beam at the sample position. This range covers most of the useful absorption edges, from the lower energies being particularly useful for sulfur SAD phasing to the chemical crystallographer’s typical Mo *K*α wavelength (0.71 Å, 17.46 keV).

The MX2 beamline features automated data processing using an in-house-developed resilient queue system based on Redis Queue (http://python-rq.org/). Collection of a data set triggers automated indexing with full integration and scaling using a modified *xdsme* (https://github.com/JunAishima/xdsme) from the original version by Pierre Legrand (https://github.com/legrandp/xdsme) and *AIMLESS* (Evans & Murshudov, 2013 ▸). *xdsme* is a Python wrapper that makes use of the programs *XDS* (Kabsch, 2010 ▸) and *Pointless* (Evans, 2006 ▸). Statistical descriptors of the data harvested from the processed data as well as collection parameters are stored on a NoSQL MongoDB database. A web client displays these descriptors with the aim of guiding strategic decisions during data collection. These systems have recently been upgraded to support the installation of the EIGER X 16M; an upgrade of the facility computing cluster is ongoing to further reduce the time it takes to fully process acquired data (complete collections or snapshots).

Routinely, data processed from MX2 has merging *R* factors in the lowest-resolution bin (below 2%) and ISa values (Diederichs, 2010 ▸) around 20 (and often above 30). Therefore, this beamline produces a high-brilliance good quality beam that is a potent tool for small or poorly diffracting crystals in need of a powerful instrument to extract data.

### 2.4. Ancillary facilities   

The beamline is supported by a physical containment level 2 certified biochemistry laboratory with fume hoods, pH meters, centrifuges, balances and other common laboratory equipment. A selection of heavy-atom and halide salts and a xenon chamber (Hampton Research) are available for derivatization of crystals at the beamline.

The control hutch is divided between an office and a small sample preparation area. The latter is equipped with two microscopes and the typical tools for tray and cryo manipulation (cryo-tongs, magnetic wands and scalpels).

The office area is equipped with five CentOS Linux computers for beamline control and computional crystallography tasks (*e.g.* manual data processing, structure solution and refinement) as well as a dual 10 Gb networked dedicated computer for data backup (to portable hard drives) and automatic loading of diffraction images using a socked based connection to *ADXV* (http://www.scripps.edu/tainer/arvai/adxv.html).

Data storage is undertaken live to two independent data storage servers for redundancy, backup and remote user access/download. After the experiment a compressed read-only single file copy in SquashFS format of the whole experiment is kept. Users can download their data remotely by an rsync mechanism, using ssh keys, as well as using SFTP, replacing the traditional hard drives at the beamline.

## 3. Facility access   

Access to beamlines at the Australian Synchrotron is *via* a merit-based scheme. Regular users access the beamlines *via* a collaborative access program whereby several laboratories group together to submit a single application for beam time to cover the whole year. International researchers or researchers who use the beamlines less frequently can apply for rapid access time and these applications can be submitted throughout the year. All applications are independently peer-reviewed and scored by the MX proposal advisory committee.

An increasingly large number of experiments on the MX2 beamline are conducted remotely, using an internet browser and the HTML5 VNC/RDP client Guacamole (https://guacamole.incubator.apache.org/). To this effect, three virtual machines provide full beamline control and crystallographic computing interface to the distant researcher *via* remote desktop.

## 4. Highlights   

The MX2 beamline is the most requested of the two MX beamlines at the Australian Synchrotron and has been involved in some exciting results from the Australasian research community. This vibrant and competitive community routinely brings increasingly challenging projects and samples to the beamline. The beamline has a strong output in high-impact science for both the biological and chemical disciplines.

### 4.1. Protein crystallography   

MX2 has been involved in elucidating important mechanisms in many different areas. Some examples in the immunology area are the structures of the mucosal-associated invariant T-cell antigen receptor in complex with the major histocompatibility complex that have shown the importance of vitamin B in cell immunosurveillance (Kjer-Nielsen *et al.*, 2012 ▸; Patel *et al.*, 2013 ▸) or the structures of T-cell antigen receptor in complex with lipids that have helped to understand how the immune system in human skin detects lipid molecules (Birkinshaw *et al.*, 2015 ▸). The latter may help in the fight against bacterial infections, cancerous cells and allergies caused by allergens such as pollen. Other examples in cell-death understanding are the structures in the Bcl-2 protein family (Czabotar *et al.*, 2013 ▸) that might lead to better future treatment for certain cancers and neurodegenerative diseases.

One such highlight is the the structure of a monoclonal antibody [solanezumab (Eli Lilly, Indianapolis, Indiana, USA)] in complex with the mid-region of the neurotoxic amyloid-β peptide which has highlighted the similarities and differences with a competing antibody [crenezumab (Genentech, South San Francisco, California, USA)] for the treatment of Alzheimer’s disease. This is particularly important when both these drugs are to be tested in multiple phase III clinical trials for the prevention of Alzheimer’s (Crespi *et al.*, 2015 ▸).

Technically challenging experiments like long-wavelength Xe anomalous phasing with pressurization carried out onsite or structure solution using molecular replacement-single-wavelength anomalous diffraction (MR-SAD), making use of the native anomalous signal from sulfur, have been performed at MX2. One such example of Xe anomalous phasing is the structure of MiD51, a receptor required for mitochondrial fission (Richter *et al.*, 2014 ▸), while examples of the use of MR-SAD are the structures of plant immune receptors RPS4 and RRS1 that helped in the understanding of the mechanism that activates the immune response to bacterial pathogens (Williams *et al.*, 2014 ▸).

**Figure 3:**
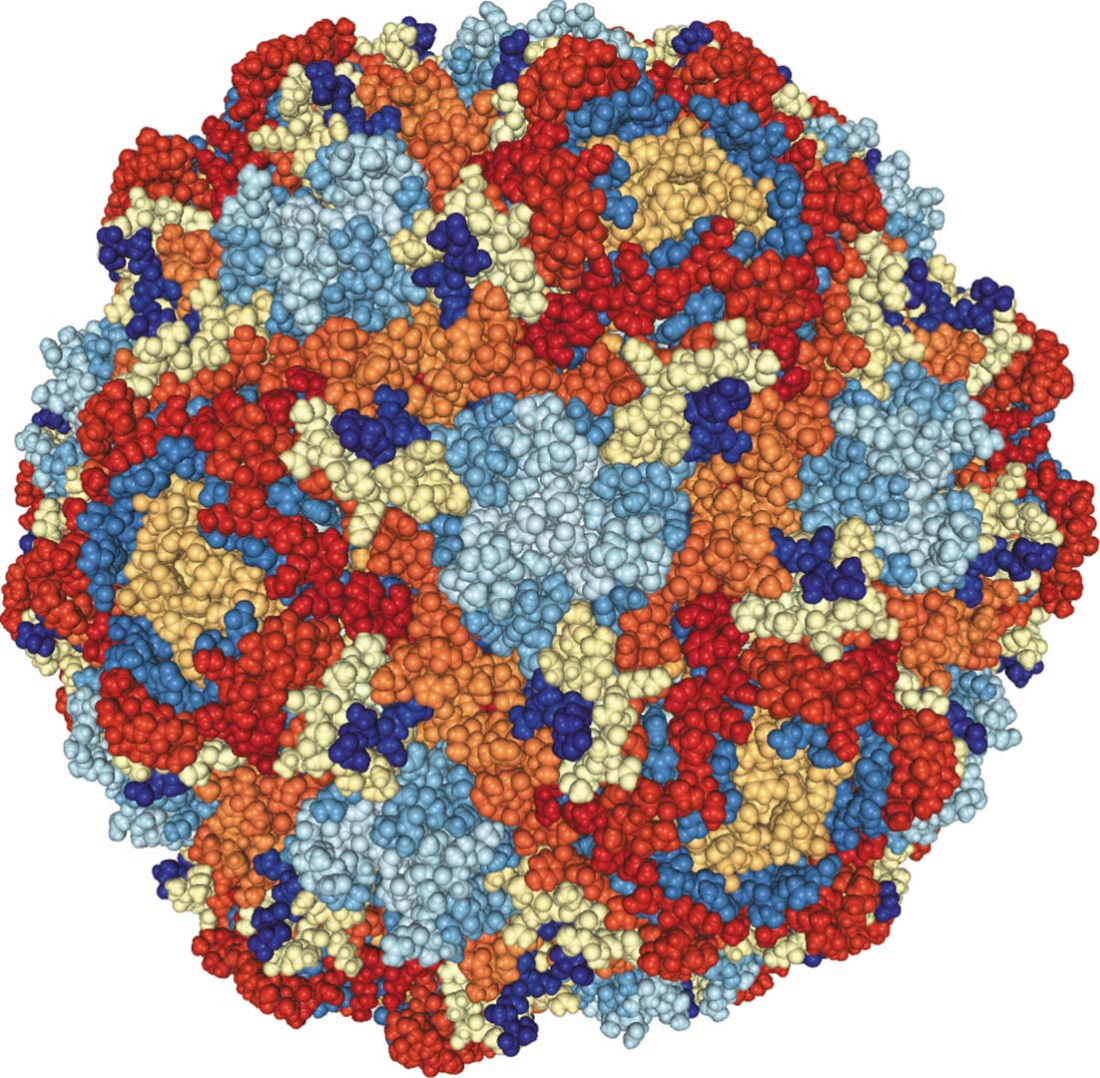
The beak and feather disease virus capsid built from 60 monomers of the capsid protein.

Other results from this beamline include insights into the structural transformations during the life cycle of pests such as the beak and feather disease virus (Sarker *et al.*, 2016 ▸). Such research might lead to a future vaccine that is becoming precious to save endangered species like the orange-bellied parrot (Fig. 3 ▸).

### 4.2. Chemical crystallography   

**Figure 4:**
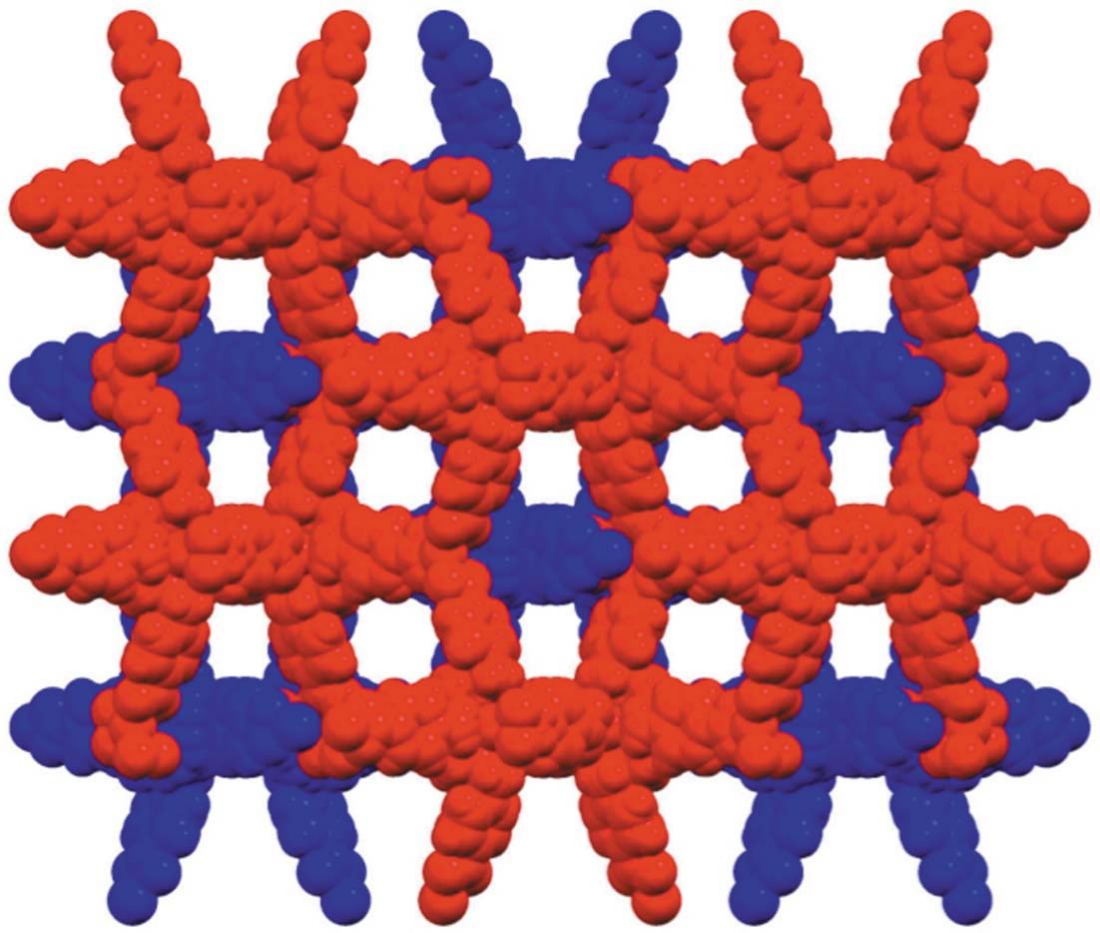
The space-filling structure of the interpenetrated porous metal organic framework (one network in red, the other in blue) showing the large void space.

There are diverse needs within the chemical crystallography community and the scope of successful publications cover micrometre-sized mineral fibres (Birch *et al.*, 2011 ▸; Grey *et al.*, 2013 ▸); strongly absorbing compounds with high-*Z* elements up to actinides (Zhang *et al.*, 2013 ▸, 2014 ▸); and weakly scattering poorly crystalline metal organic frameworks with large and dis­ordered solvent void spaces. One example of the latter is the use of metal organic frameworks (MOFs; Fig. 4 ▸) for the separation of complex mixtures of polyaromatic hydro­carbons and polar analytes which has shown the potential for MOFs to be utilized in real world separations of complex mixtures (Hawes *et al.*, 2014 ▸). The MOF is assembled from a cyclen-based cadmium complex with four benzoate pendant arms allowing for the formation of an extended framework. The use of a microfocus high-intensity beamline like MX2 has proved invaluable to overcome the tendency for MOFs to have poor crystalline order and large void spaces occupied with disordered solvent which leads to strong falloff in diffraction at high resolution as well as diffuse scatter from the poorly ordered solvent molecules.

Other recent results from this beamline include investigations of novel liquid crystal materials like benzodithiophene terthiophene rhodanine which may lead to better solar panels in the future (Sun *et al.*, 2015 ▸).

## 5. Discussion and conclusions   

The MX2 beamline is the workhorse for the crystallographic communities in Australia and New Zealand both in terms of the number of structures solved and producing the highest-impact publications. It caters not only for the chemical and protein crystallography communities but also specifically for those in need of high flux and microbeam technology. Being the most subscribed and most used of the two MX beamlines, both user demand and wear and tear are driving recent and future hardware and software upgrades. In the next six months there is a plan in place to upgrade the sequence of motions of the robot which will increase the speed of the process and should reduce the sample to sample time to less than 30 s (Russi *et al.*, 2016 ▸). The plan also incorporates replacing the mirror substrates that are showing significant wear and tear and installing new computer hardware to process EIGER data. On a longer timescale we are also aiming to replace *Blu-Ice* with a more modern web-based beamline control UI.

The high-flux and specialist features of this beamline make it particularly useful for the most difficult crystallographic projects. A vibrant user community, with complex biological problems to be solved, has raised the bar creating a continuous state-of-the-art research output.

## Supplementary Material

Supporting Figure S1. DOI: 10.1107/S1600577518003120/ig5051sup1.pdf

## Figures and Tables

**Table 1:** Summary of the beamline hardware for MX2

Beamline name	Micro Crystallography – MX2
	
Source type	U22 undulator
Total length	3 m
Period	22 mm
Minimum gap	6.6 mm
Peak field @ minimum gap	0.85 T
*K*_max_	1.75
Monochromator	Double-crystal Si(111) liquid nitrogen cooled (DC) or channel-cut Si(111) liquid nitrogen cooled (CC)
	
Energy range (keV)	
User controlled (DC)	8.0–15.5
Full range available (DC)	6.5–18.0
User controlled (CC)	12.0–13.5
	
Wavelength range (Å)	
User controlled (DC)	1.55–0.80
Full range available (DC)	0.69–1.91
User controlled (CC)	0.92–1.03
	
Mirrors	Three Si mirrors with Rh and Pt stripes; one vertical focusing (VFM), one horizontal focusing (HFM) and one horizontal microfocusing (MHFM). All mirrors operate at or close to 2.8 mrad incident angle.
	
Beam size, without apertures (FWHM) (H × V) µm	22 × 12 µm
	
Photon flux (at 13 keV)	
Full beam	3.4 × 10^12^ photons s^−1^
20 µm aperture	5.1 × 10^11^ photons s^−1^
10 µm aperture	2.0 × 10^11^ photons s^−1^
7.5 µm aperture	1.4 × 10^11^ photons s^−1^
	
Goniometer	Horizontal air-bearing
Cryo capability	CryoJet 5
Pixel detector†	EIGER X 16M (Dectris Ltd, Switzerland)
Fluorescence detector	Vortex Si-drift detector (Hitachi High-Technologies Science America, Chatsworth, CA, USA)
Sample mounting	SAM system

† From February 2017.
