# Supplementary material for: MX2: a high-flux undulator microfocus beamline serving both the chemical and macromolecular crystallography communities at the Australian Synchrotron
Source: J Synchrotron Radiat. 2018 Apr 3;25(Pt 3):885–91. doi: 10.1107/S1600577518003120 (PMC5929359; doi:10.1107/S1600577518003120)
Supplement: Supplementary file 1 [file s-25-00885-sup1.pdf]

JOURNAL OF SYNCHROTRON RADIATION

Volume 25 (2018)

Supporting information for article:

MX2: A high-flux undulator microfocus beamline serving both the chemical and macromolecular crystallography communities at the Australian Synchrotron

David Aragão, Jun Aishima, Hima Cherukuvada, Robert Clarken, Mark Clift, Nathan Philip Cowieson, Daniel Eriksson, Christine L Gee, Sofia Macedo, Nathan Mudie, Santosh Panjikar, Jason Roy Price, Alan Riboldi-Tunnicliffe, Robert Rostan, Rachel Williamson and Thomas Tudor Caradoc-Davies

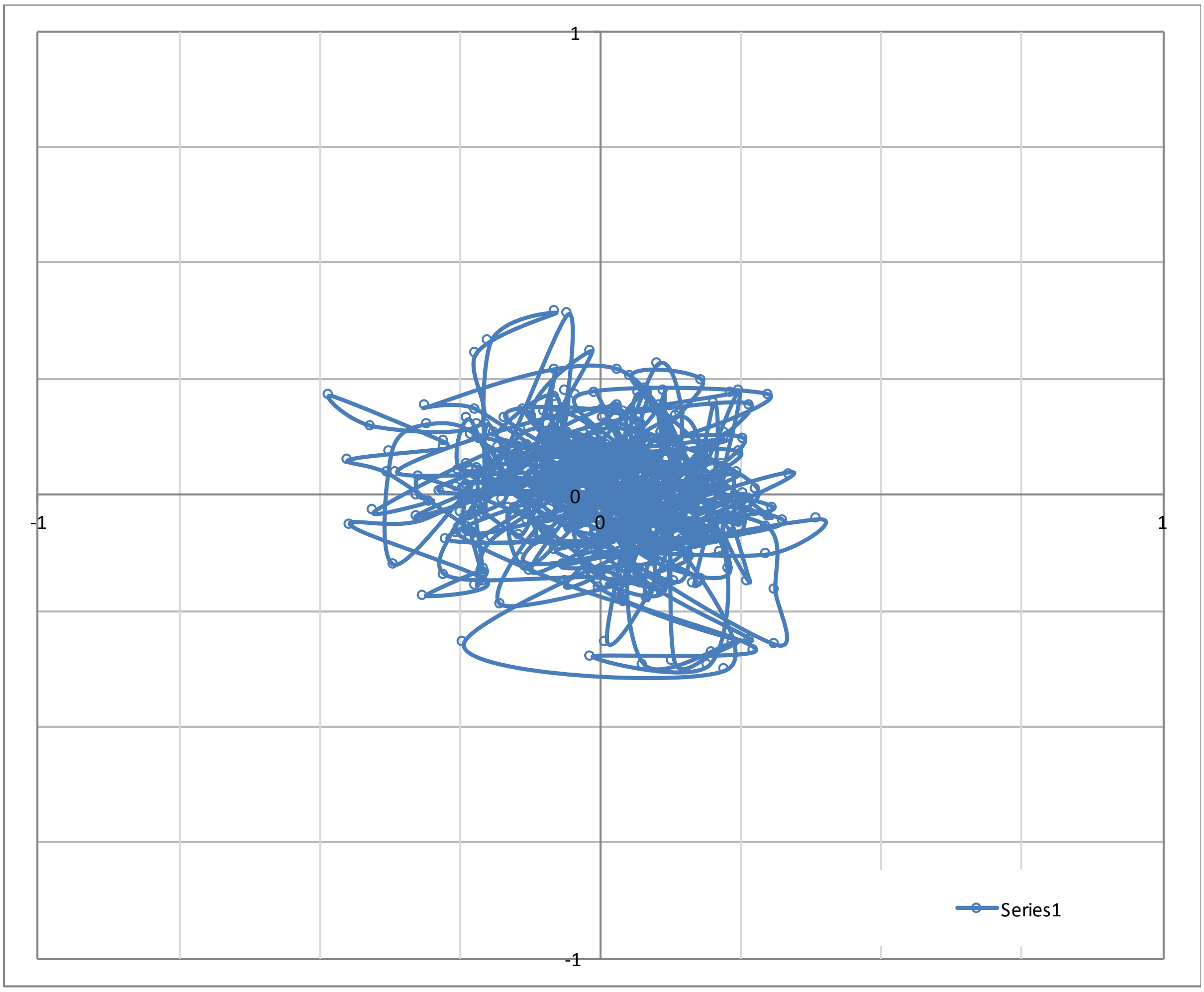

Figure S1 Sphere of confusion analysis on goniometer using laser interferometer while rotating the goniometer at 50 degrees per second for 6 full 360 rotations. This results on a sphere of rotation of ± 0.33 µm in horizontal and ± 0.26 µm on the vertical axis.
